# Relation of Hemorrhoidal Disease to the Health Balance

**Published:** 1917-12

**Authors:** William M. Beach

**Affiliations:** Pittsburgh, Pa.


### RELATION OF HEMORRHOIDAL DISEASE TO THE HEALTH BALANCE.

**By William M. Beach, A.M., M.D., F.A.C.S., Pittsburgh, Pa.**

In discussing the "Relation of Hemorrhoidal Disease to the Health Balance" Beach sums up as follows:

1. The deleterious effect upon the patient's mind by increasing his irritability and making him anxious and morose.

2. The many reflexes coincident with ulcerated large or small type of hemorrhoids.

3. The influence upon the so-called vegetive functions of the body and intimate associations with diseases of the heart, lungs, liver and kidneys.

4. The refractory or retroactive relationship in most cases of constipation.

5. The tendency of neglected cases toward infections and cancer.

